# Using bear rub data and spatial capture-recapture models to estimate trend in a brown bear population

**DOI:** 10.1038/s41598-019-52783-5

**Published:** 2019-11-14

**Authors:** Katherine C. Kendall, Tabitha A. Graves, J. Andrew Royle, Amy C. Macleod, Kevin S. McKelvey, John Boulanger, John S. Waller

**Affiliations:** 1 U.S. Geological Survey, Northern Rocky Mountain Science Center, West Glacier, Montana 59936 USA; 2 Present Address: Ursine Ecological, Columbia Falls, Montana 59912 USA; 3 U.S. Geological Survey, Northern Rocky Mountain Science Center, West Glacier, Montana 59936 USA; 4 U.S. Geological Survey, Patuxent Wildlife Research Center, Laurel, Maryland 20708 USA; 5 Applied Conservation Ecology, University of Alberta, Edmonton, Alberta T6G 2H1 Canada; 6 U.S. Forest Service, Rocky Mountain Research Station, Missoula, MT 59801 USA; 7 Integrated Ecological Research, Nelson, British Columbia V1L 5T2 Canada; 8 U.S. National Park Service, Glacier National Park, West Glacier, Montana 59936 USA

**Keywords:** Statistical methods, Population dynamics, Animal behaviour

## Abstract

Trends in population abundance can be challenging to quantify during range expansion and contraction, when there is spatial variation in trend, or the conservation area is large. We used genetic detection data from natural bear rubbing sites and spatial capture-recapture (SCR) modeling to estimate local density and population growth rates in a grizzly bear population in northwestern Montana, USA. We visited bear rubs to collect hair in 2004, 2009—2012 (3,579—4,802 rubs) and detected 249—355 individual bears each year. We estimated the finite annual population rate of change 2004—2012 was 1.043 (95% CI = 1.017—1.069). Population density shifted from being concentrated in the north in 2004 to a more even distribution across the ecosystem by 2012. Our genetic detection sampling approach coupled with SCR modeling allowed us to estimate spatially variable growth rates of an expanding grizzly bear population and provided insight into how those patterns developed. The ability of SCR to utilize unstructured data and produce spatially explicit maps that indicate where population change is occurring promises to facilitate the monitoring of difficult-to-study species across large spatial areas.

## Introduction

Understanding trends in population abundance is fundamental to wildlife management and can be critically important for conservation of at-risk taxa^1^. Trend can be particularly challenging to quantify when population density is low, the population and its range are expanding or contracting, there is spatial variation in trend within the population, or the conservation area is large or remote; traits common to most forest carnivore populations. Grizzly bears (brown bear; *Ursus arctos*) epitomize the characteristics that make populations difficult to monitor. They occur at low densities^2^, are solitary (except while females are accompanied by their young), have large home ranges^3^, often move long distances in short periods of time^4^, can exhibit strong behavioural responses to sampling that employs consumable baits and handling, and tend to only survive in remote areas^5^. Many populations are in decline throughout their Holarctic distribution but some have begun to recover and reoccupy former range in recent years^6–10^. Estimating bear density can be difficult near such expansion fronts because large differences in densities can occur over short distances and may require fine-scale sampling over large areas^11^.

Bears routinely rub vigorously on trees and other objects, often leaving hair behind. This ubiquitous activity is most likely a form of marking behaviour that functions as chemical signaling to other bears^12,13^. By exploiting natural rubbing behaviour, it is possible to detect a substantial portion (36%^8^ to 85%^13^) of a bear population without the use of bait. Hair samples found at bear rub sites usually have sufficient DNA for genetic determination of species, sex and individual identity^13–16^. Thus, periodic hair collection from rub objects produces a spatial array of repeated detections for individual bears appropriate for capture-recapture modeling^17^. When detection is imperfect, capture-recapture modeling provides an optimal approach for estimating population size, trend, and other demographic parameters.

The Northern Continental Divide Ecosystem (NCDE) in northwestern Montana, USA, is home to one of five North American grizzly bear populations persisting south of Canada. All were designated threatened in 1975^5^. Bear density in the NCDE in 2004 was highest in Glacier National Park (NP) in the north with density declining to the south and to the edges of occupied range^8,18^. Along the southern boundary of Glacier NP, the presence of a busy highway (US 2) impacts bear crossings^19^. Gene flow is reduced across the corridor that contains the western portion of US 2 and the rail line that parallels it^8^. Population size in the NCDE was estimated to have increased from 765 in 2004^8^ to approximately 1,000 by 2009^20^. Because this trend estimate was based on a small portion (2–4%) of the population, which increases the potential for bias, we sought to increase the proportion sampled and improve insight on population status by detecting individual bears from hair at natural bear rub sites. The purpose of our study was to explore this new approach to monitoring the trajectory of a low-density carnivore population at an ecosystem scale and to estimate variation in local density and growth rates within the population using a spatial capture-recapture modeling framework.

## Methods

### Field methods

**Figure 1:**
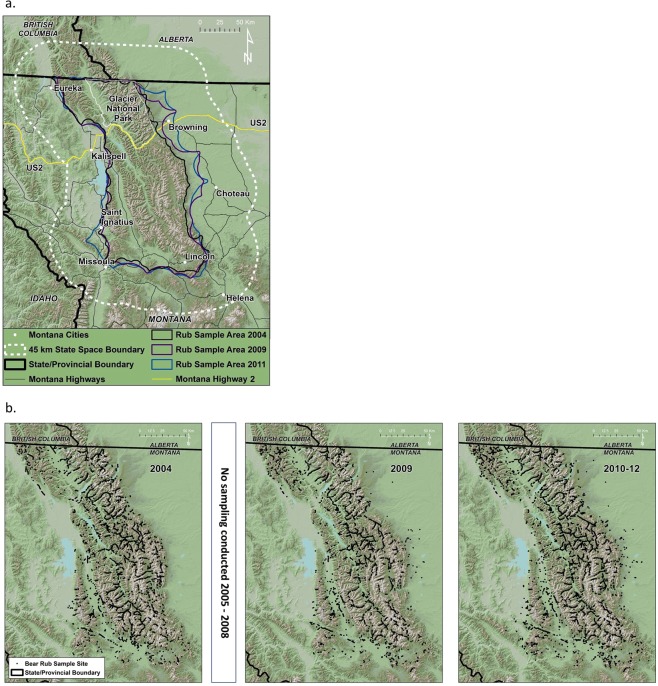
(**a**) Northern Continental Divide Ecosystem study area in northwestern Montana, USA where growth rates of an expanding grizzly bear population were estimated during 2004–2012. State-space specified for the spatial capture-recapture models extended 45 km beyond the area where bear rubs were sampled. (**b**) Locations of naturally-occurring bear rub sites where the grizzly population was sampled by collecting hair for genetic determination of individuals 2004, 2009, 2010–2012. Maps were created from the USGS National Elevation Dataset^58^ using ArcMap 10.2.

We sampled the NCDE grizzly bear population in a 33,300-km^2^ area dominated by the rugged and remote terrain of the Rocky Mountains (Fig. 1a). We collected hair at bear rubs sites identified by looking for the presence of hair on trees and other objects while searching along trails, roads, and utility and fence lines. Hair deposition was a result of natural behaviour; no attractant was used to draw bears to survey routes or to encourage rubbing. Rubs were fitted with 3 to 4 40-cm lengths of 4-point barbed wire to facilitate hair deposition^8^. We monitored the rubs we located in 2004 and 2009–2012 (see Supplementary Information online for additional details).

We quantified rub sampling effort as the cumulative number of days between the first collection visit (after the initial visit to clear hair that may have been deposited the previous year) and last collection of the year for each rub sampled per year (Table 1). Sampling began in early June in all years, ended in early September in 2004, and most sampling ended in early October 2009–2012.

**Table 1:** Sampling effort and number of individual grizzly bears detected at natural bear rub sites monitored in the Northern Continental Divide Ecosystem in northwestern Montana, USA during 2004, 2009–2012.

Year	Rub sites	Sampling occasions	Cumulative rub sampling days^a^	Individual grizzly bears detected
2004	4,802	4	312,882	274
2009	3,579	2	289,822	249
2010	4,608	2	344,942	334
2011	4,711	2	342,157	321
2012	4,635	2	342,972	355

The number of rub sites monitored only includes those visited ≥ 1 time after the initial visit to remove hair that could have been deposited the previous year.

^a^Cumulative number of days between the first visit and last collection of the year for each rub sampled per year. For example, if we surveyed 1,000 rubs 1 time after surveying them 60 days earlier, and sampled 2,000 rubs 2 times at 45-day intervals and 100 rubs 3 times at 40-day intervals, rub sampling effort for that year would be (1,000 × 60) + (2,000 × 45 × 2) + (100 × 40 × 3) = 60,000 + 180,000 + 120,000 = 360,000 rub sampling days. Sampling days began to accrue after the initial “clean-off” visit to each rub each year.

### Genetic analysis

Hair samples were analyzed by a laboratory that specialized in genotyping low quantity and quality DNA (Wildlife Genetics International; http://www.wildlifegenetics.ca). We used the G10J marker to distinguish between grizzly bear and black bear (*U. americanus*) samples^21^. We added 6 additional nuclear microsatellite loci to determine individual identity of the grizzly bear hair samples (G1A, G10B, G1D, G10H, G10M, and G10P)^22–24^ and the amelogenin marker to identify sex^25^. We confirmed individuals identified using this 7-locus genotype by extending the genotypes to 9 additional loci (G10C, G10L, CXX110, CXX20, Mu50, Mu59, G10U, Mu23, and G10X^22,23,24,26,27^, and confirmed sex with a second proprietary test developed by Wildlife Genetics International. Sub-selection of samples for analysis is described in Supplementary Information online.

### Spatial capture-recapture modeling

We used maximum-likelihood spatial capture-recapture (SCR) models^28,29^ to estimate local and overall density and annual population growth rates from spatial detection histories. SCR models specify a spatially explicit link between a summary of each individual’s location, henceforth their activity center, and locations where they may be detected, here, the sampled bear rubs. We treated the 5 years (2004, 2009, 2010, 2011, 2012) of detection data as independent “occasions” and ran separate models for males and females. For each year we denoted the coordinates of each rub by the vector *x*_*jt*_ for years t = 2004 and 2009–2012 and for j = 1, 2, …, *n*_*t*_ rub locations and the activity center for individual *i* as *s*_*i*_, a 2-dimensional coordinate. SCR models parameterize the probability of detection, *p*(*x*_*jt*_,*s*_*i*_), of an individual at each rub by a function of distance between the rub and the putative activity center. For each year and sex, we used the half-normal model^30^, where probability of detection at a location is assumed to decline with distance from the activity center following a half-normal distribution such that:

1 $$p({x}_{jt},{s}_{i})={p}_{0}exp(-dist{({x}_{jt},{s}_{i})}^{2}/(2{\sigma }^{2}))$$

The parameters we estimated were baseline detection probability at the activity center, *p*_0_, and the scale of movement parameter, σ, related to the extent of space used by individuals during the period of sampling. In addition, SCR models contain a parameter representing density, *D*, estimated from the observed encounter history data. Model parameters were estimated by maximum likelihood (MLE^31^) using the R package oSCR^31,32^. For all parameters of the model the standard error (SE) was from the Hessian of the likelihood function evaluated at the MLEs.

We developed our model set based on our *a priori* knowledge of grizzly bear ecology and theories about factors influencing bear behaviour. The hypotheses we considered modeled various effects on each of the 3 primary parameters; density (*D*), baseline detection probability (*p*_0_), and the scale of movement parameter (*σ*, Table S1 online). Effects were modeled on each parameter on a link-transformed scale, with an intercept and effects modeled additively. We modeled temporal variation in *D* by allowing full year-specificity, *log*(*D*_*t*_) = *β*_t_, or a linear trend *log*(*D*_*t*_) = *β*_0_+*β*_1_*Year* where *Year* was the centered year (2008 = 0). Density and *σ* were modeled on the log-scale and baseline detection probability was modeled on the logit scale. We included duration of sampling interval, linear effects of date, a trap-specific behavioural response, and landscape covariate effects in all models for *p*_0_ and evaluated hypotheses that included year-specific and quadratic effects of Julian date. We fit a behavioral model because rubbing functions as communication among individuals and thus prior marking at a rub may change the likelihood that site will be rubbed again by that or other individuals in the future. We calculated rub-specific landscape detection covariates to describe ruggedness, security level (protected land ownership), and the linear and quadratic effects of percent canopy cover within 250-m of a rub^33^ (see Supplementary Information online for additional details). We added these covariates to test the hypotheses that detection would be higher; 1) in rugged areas because bears might be more likely to walk on trails, 2) where there was higher security, and 3) where trees were of moderate to high density. We included hypotheses that *σ* was constant or year-specific. We used the sample size-adjusted Akaike’s Information Criterion (AIC_*c*_) and AIC_*c*_ weights^34^ to evaluate relative support for each candidate model. Models with cumulative weights up to 1.0 were considered supported.

To address human-bear conflicts, wildlife managers sometimes move bears large distances resulting in unnaturally large distances between detections^35^. To assess the impact of this on our density estimates, we used the best model for each sex from the initial set of candidate models, but allowed *σ* and *p*_0_ to vary by translocated individuals. These models would thus estimate the proportion of translocated individuals, defined as *Ψ*_*trans*_ = *Pr*(translocated = 1)^36^.

To calculate trend, we used the most supported models. For models varying linearly with year, the trend parameter was estimated directly within the model. For models with year-specific density parameters, we characterize trend by the annual geometric mean rate of population change^37^, λ, from 2004–2012 (8 annual intervals) according to:

2 $$\lambda =({D}_{{\rm{2012}}}/{{\rm{D}}}_{2004})\wedge (1/8)$$

The MLE of λ was obtained by plugging in the MLEs of year-specific densities. The standard error (SE) of estimated rates of population change was computed using a delta approximation^38^. The SEs were used to obtain 95% Wald type confidence interval. These growth rate estimates apply to the entire state space. We also compared our estimates to the trend in effective population size, N_e_, a metric referring to the number of bears that are contributing reproductively to the population, under a set of genetic assumptions. For this we used analysis of covariance as described in Pierson *et al*.^39^.

In SCR models, MLEs are based on the marginal likelihood of the encounter histories computed by averaging over all possible locations of the activity centers associated with detected individuals. This spatial region is called the “state space”. The state-space should be chosen to be sufficiently large such that an individual having an activity center on the periphery of the state-space has negligible probability of capture (^40^ p. 132). For the half-normal detection model we used, we concluded that 3 σ was sufficiently close to negligible, yielding a probability of encounter < 0.01 based on estimates from our model. In our initial model runs, male σ was 15 km so we defined our state space by a regular grid of points (4 km grid spacing) extending 45 km beyond any rub location (Fig. 1). The number of state-space points varied with locations of rubs monitored each year and ranged from 3,992 (2004) to 4,422 (2012). SCR models regard the activity center, *s*_*i*_, as a latent variable that is estimated along with other parameters. We estimated the latent activity centers using the estimated best unbiased predictor^41^ which is the conditional mean of *S*_*i*_ given the data, evaluated at the MLEs. Predicted density surfaces were generated by aggregating the estimated posterior distribution of each *S*_*i*_. To examine changes in local trend over time, we estimated the realized local population size (aka realized density) for each state-space point (^40^ sec. 5.11), using the MLEs of the model parameters. The estimated realized population size for any state-space point **u** is defined as the estimated posterior mean of the number of activity centers associated with point **u**. This is computed by adding (a) the sum of the posterior probability of each observed individuals’ activity center for **u** to (b) the expected number of individuals for that state-space point that went undetected. The former quantity, (a), is denoted by $Pr({{\bf{s}}}_{i}={\bf{u}}|{{\bf{y}}}_{i},\theta )$ (^40^ ch. 6) where $\theta$ represents the collection of all model parameters for a given model, and we plug-in the MLEs for $\theta$, $\hat{\theta }$ for this evaluation. Component (b) is denoted by ${n}_{0}({\bf{u}}|\theta )$ which is also a function of all model parameters (^40^ ch. 6). Thus, the expression for the estimated realized population size for any point **u** of the discrete state-space is given by:

3 $$\hat{D}({\bf{u}})=\{\mathop{\sum }\limits_{i=1}^{n}Pr({{\bf{s}}}_{i}={\bf{u}}|{{\bf{y}}}_{i},\hat{\theta })\}+E({n}_{0}({\bf{u}}|\hat{\theta }))$$

This calculation can be done using the model parameters for each year to produce ${\hat{D}}_{t}({\bf{u}})$, from which estimated local growth rates can be computed for each unit of the state space,

4 $$\lambda ({\bf{u}})={(\frac{{\hat{D}}_{2012}({\bf{u}})}{{\hat{D}}_{2004}({\bf{u}})})}^{1/8}.$$

Local growth-rate estimates were limited to the area that was sampled in 2004 as this area was sampled in all years. We also calculated growth rates above and below Highway 2, a known barrier to gene flow^8^, by summing the estimated density of the state space points within each region in each year and calculating λ from those regional estimates. Variance estimates are not available for these sub-zones, however, because variance was calculated for the full state space and cannot be partitioned.

In one year, we detected one female bear 190 km to the south of other detections of this individual. Although grizzly bears are capable of traveling long distances, having a female range this far between detections, i.e. almost 3 times the distance of the second largest female movement detected (64 km), was highly unusual and constituted the lone extreme movement in our data set. While we did not identify any errors that could explain this large movement and it was not associated with a known translocation, in spatial modeling it is common to remove outlier locations when they are not representative of the population and leverage results^42^. We, therefore, fit all hypothesized models with and without the southern location for this individual, but consider primary results to be those associated with models with the location removed. Results from complete models that include this location are presented in the online Supplementary Information.

## Results

### Field and genetic

We monitored 3,579–4,802 bear rubs annually in the NCDE in 2004, 2009–2012 (Table 1, Fig. 1b). The distribution of rubs we sampled varied between 2004, 2009 and 2010 but was nearly identical 2010–2012 (Fig. 1). See Supplementary Information online for an in-depth explanation of differences in sampling effort and extent. Despite expanding sampling east of the mountains after 2004, few bears were detected there suggesting the area was minimally occupied in 2004 (Figure S1 online). Rubs were visited on at least one occasion annually to collect hair; median = 4 visits/rub in 2004, 2 visits/rub 2009–2012, not including an initial visit to each rub to clear hair that could have been deposited the previous year (Table 1). We detected 249–355 individual grizzlies each year for a total of 394 different females and 398 males during our 5 sampling years (Table 2). Our laboratory protocols made it highly unlikely that genotyping error resulted in the creation of any false detections in our data^8^.

**Table 2:** Number of female and male grizzly bears detected at bear rub sampling sites in northwest Montana 2004, 2009–2012.

Year	Individuals			Detections			
	Detected	Recaptured	Spatially recaptured	Total	Spatial	MMDM	Max Distance
**Females**							
2004	119	57	55	216	205	6.49	43
2009	94	31	29	138	136	7.16	64
2010	145	70	67	285	273	5.12	17
2011	143	55	54	257	248	5.27	30
2012	167	68	68	303	293	5.56	38
**Males**							
2004	155	97	95	668	626	18.43	113
2009	154	93	93	505	495	16.01	68
2010	189	103	101	554	540	17.90	143
2011	178	103	102	497	486	15.86	60
2012	188	111	111	532	523	16.68	104

Individual recaptures are subsequent detections of an individual at the same or new locations. Spatial recaptures of an individual are detections in locations different from the initial and subsequent detections. The number of total detections is the total number of times bears were detected, e.g. in 2004, 119 bears were detected 216 times. Spatial detections count only detections in different locations, e.g. in 2004, 119 bears were detected in 205 unique locations. Mean maximum distance moved (MMDM) is mean distance between detections for each individual identified averaged for all bears. Max distance is the longest distance between 2 detection locations for any individual. Summary does not include 1 detection of a female in 2010 that was 190 km from other detections of that individual that year.

### Spatial modeling

Our models were remarkably sensitive to the single extreme movement between detections in our data. Removal of this point in 2010 changed the most supported female models from those with year-specific density to models with a linear trend in density (compare Table S3 online, Table 3). However, estimated female population growth rates were similar: λ_04–12_ was 1.042 (CI: 1.023–1.060) versus 1.050 (95% CI: 1.010–1.089) with and without the outlier, respectively. Estimates differed primarily in 2010. Namely, σ was much larger and baseline detection and density were much smaller in 2010 than other years (Table S4 online). For the top model, estimates of density were significantly lower and estimates of σ were significantly higher than estimates without the outlier location (non-overlapping CI’s: Table S4, S5 online). When the outlier point was excluded, density estimates in 2010 were more consistent with estimates for the other years.

**Table 3:** Description of most supported models (models with the lowest AIC scores with cumulative weights up to 1.0) developed to estimate grizzly bear population density using spatial capture-recapture data from the Northern Continental Divide Ecosystem in northwestern Montana, USA.

Model^a^	D	*p*0	σ	ΔAIC	Weight	CumWt
**Females**						
1	~linear	~year + dur + jul + jul2 + beh + spatial covs	~year	0.00	0.61	0.61
2	~linear	~year + dur + jul + beh + spatial covs	~year	1.26	0.32	0.93
3	~year	~year + dur + jul + jul2 + beh + spatial covs	~year	5.24	0.04	0.98
4	~year	~year + dur + jul + beh + spatial covs	~year	6.51	0.02	1.00
**Males**						
1	~linear	~year + dur + jul + jul2 + beh + spatial covs	~year	0.00	0.95	0.95
3	~year	~year + dur + jul + jul2 + beh + spatial covs	~year	5.80	0.05	1.00

Spatial detection histories were derived from genetic identification of individual bears from hair collected at natural bear rub sites. For females, a single outlier point in 2010 was removed from the dataset. See Table S1 online for descriptions of the complete set of candidate models.

^a^Model notation: D: density (bears/1,000 km2). p0: baseline detection probability. σ: sigma; spatial scale parameter related to the amount of space used by each individual. ΔAIC: cumulative change in Akaike Information Criterion. Weight: measure of support for each model. CumWt: cumulative measure of support for the models. ~: “a function of”. linear: density is a linear function of time. year: parameter is year specific. dur: duration of sampling interval; number of days since previous sampling visit. jul: Julian date; linear effect of season on detection probability. jul2: Julian date squared; quadratic effect of season on detection probability. beh: behavioral response; a visit of some bear at some bear rub changes its subsequent probability of detection at that rub. spatial covs: spatial covariates: cover: percent of mean canopy cover within 250 m of the trap. cover2: percent of mean canopy cover squared. curv: standard deviation of terrain curvature within 250 m of the trap. sec: security level as determined by land ownership policies at the trap: 10 = U.S. National Park Service, 7 = U.S. Forest Service, 3 = state/other public, 1 = private).

Because this one long-range movement was not representative of the population and had such a large effect, we focus here on model results that did not include this data point. The most supported model by far for females and males defined density as increasing linearly with year, year-specific σ, and included all detection covariates (Table 3; see Table S1 online to view all candidate models). Detection increased with duration, lower canopy cover, and a higher percent of secure area, and had a quadratic function for Julian date (Table S2). Models with a translocation effect did not converge, likely due to low sample size of translocated bears within the DNA data set (12 females, 9 males).

**Figure 2:**
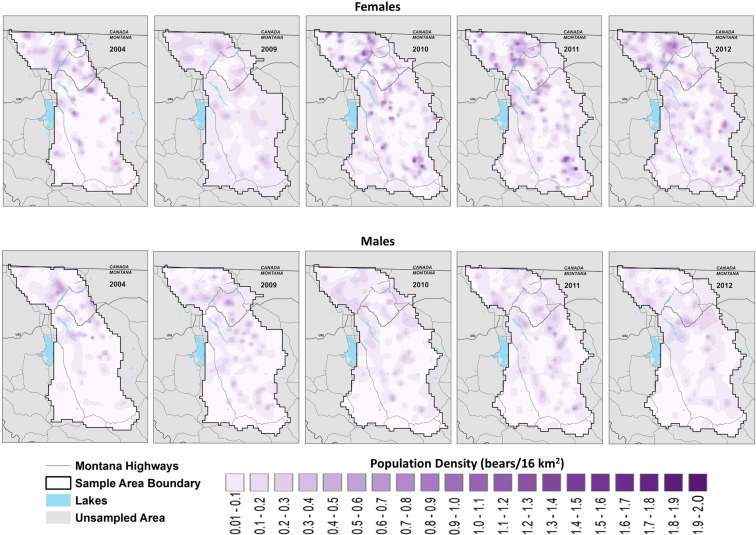
Annual predicted density surfaces of the grizzly bear population in the Northern Continental Divide Ecosystem 2004, 2009–2012. Density estimates (bears/16 km^2^) were based on the most supported spatial capture-recapture model of detection histories at natural bear rub sites. We limited estimates to the area in which rubs were identified and sampled, which varied by year.

Annual estimates of density were similar among all supported models (Table 4). Both the size and distribution of the grizzly bear population in the NCDE expanded between 2004 and 2012. Ecosystem-wide, female density increased from 7.77 bears/1,000 km^2^ (95% CI: 6.37–9.48) in 2004 to 11.45 bears/1,000 km^2^ (95% CI: 9.80–13.38) in 2012. Male density rose from 5.78 bears/1,000 km^2^ (95% CI: 4.96–6.72) in 2004 to 7.51 bears/1,000 km^2^ (6.81–8.27) in 2012. In 2004, the majority of the population resided in the northern third of the ecosystem centered on Glacier NP. As the population grew, density increased throughout the ecosystem with the most obvious changes in the southern two thirds of the NCDE where bears were absent or density was low in 2004 (Fig. 2).

**Table 4:** Estimates (transformed) of density, D, and spatial scale of movement, σ, parameters for models developed to estimate grizzly bear population trend using spatial capture-recapture data from the Northern Continental Divide Ecosystem in northwestern Montana, USA.

	Model	Female				Male	
		1	2	3	4	1	3
D (95% CI) Bears/1,000 km^2^	**Year**						
	2004	7.77 (6.37–9.48)	7.76 (6.37–9.47)	7.92 (6.42–9.77)	7.90 (6.41–9.75)	5.78 (4.96–6.72)	5.75 (4.89–6.75)
	**No sampling 20005–2008**						
	2009	9.90 (9.00–10.90)	9.91 (9.00–10.90)	8.39 (6.32–11.15)	8.40 (6.33–11.16)	6.80 (6.34–7.30)	6.77 (5.74–7.97)
	2010	10.40 (9.34–11.57)	10.40 (9.35–11.57)	10.16 (8.39–12.29)	10.18 (8.41–12.32)	7.03 (6.54–7.56)	7.20 (6,20–8.34)
	2011	10.91 (9.60–12.40)	10.92 (9.61–12.41)	10.39 (8.51–12.68)	10.38 (8.50–12.67)	7.27 (6.69–7.88)	7.32 (6.27–8.53)
	2012	11.45 (9.80–13.38)	11.47 (9.81–13.40)	11.04 (9.21–13.22)	11.04 (9.21–13.23)	7.51 (6.81–8.27)	7.36 (6.33–8.54)
σ (95% CI) km	2004	4.40 (3.96–4.88)	4.39 (3.95–4.87)	4.39 (3.96–4.87)	4.38 (3.95–4.86)	9.30 (8.81–9.81)	9.30 (8.81–9.81)
	**No sampling 2005–2008**						
	2009	5.84 (4.99–6.83)	5.83 (4.98–6.82)	5.89 (5.01–6.92)	5.88 (5.00–6.90)	7.34 (6.92–7.78)	7.35 (6.93–7.79)
	2010	3.05 (2.79–3.33)	3.05 (2.79–3.33)	3.04 (2.78–3.33)	3.04 (2.78–3.33)	9.17 (8.65–9.72)	9.15 (8.63–9.71)
	2011	3.24 (2.94–3.57)	3.24 (2.94–3.58)	3.24 (2.94–3.58)	3.24 (2.94–3.58)	7.62 (7.16–8.10)	7.62 (7.16–8.10)
	2012	3.86 (3.53–4.22)	3.86 (3.53–4.22)	3.86 (3.53–4.22)	3.86 (3.53–4.22)	9.12 (8.59–9.68)	9.13 (8.59–9.69)

**Figure 3:**
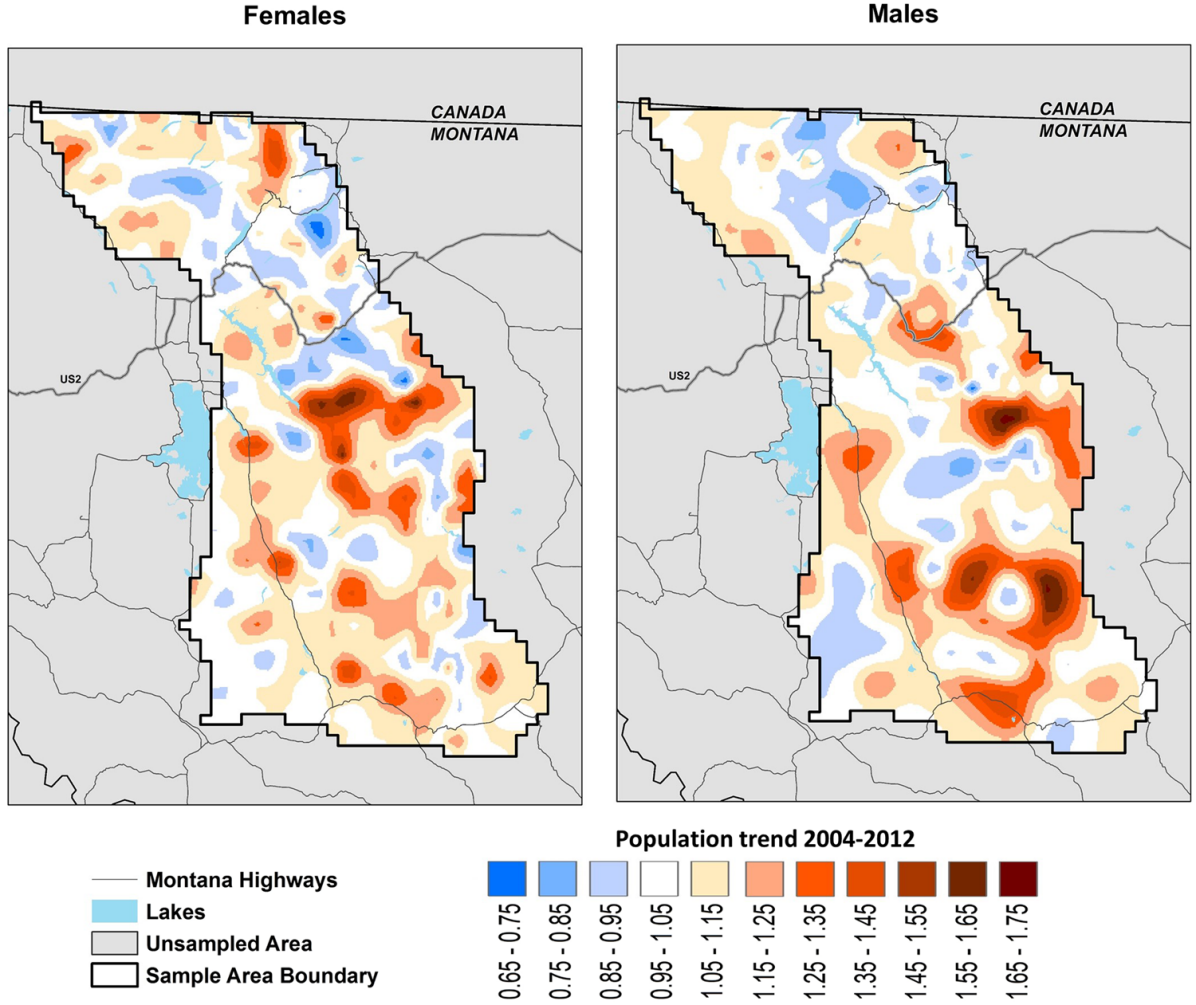
Annual local rates of change in grizzly bear population density within the Northern Continental Divide Ecosystem in northwestern Montana 2004–2012. Population trend was calculated as the annual geometric rate of change in grizzly bear density (*D*) between 2 years. Rate of population change was calculated for each 16 km^2^ state space pixel defined by our spatial capture-recapture model. The estimate area was smaller for 2004 than in subsequent years because few bear rubs were found along the eastern edge of the study area that year. Local density should only be inferred for the area sampled. Growth rates > 1.0 denote areas of population growth; rates < 1.0 denote areas of population decline.

The annual rate of overall population change 2004–2012 (λ_04-12_) was 1.043 (95% CI: 1.017 1.069). The growth rates of the female and male segments of the population were not significantly different; λ_female04-12 = _1.050 (95% CI: 1.010–1.089), λ_male04-12_ = 1.033 (95% CI: 1.007–1.060) (Table 5). Both female and male growth rates were higher south of US 2 (F: 1.051, M: 1.053) than north (F: 1.030, M: 1.010). Most of the ecosystem exhibited stable to increasing local population trends, however, some areas experienced declines for one sex or the other (Fig. 3).

**Table 5:** Population growth rate estimates, λ, 2004–2012 for the most supported spatial capture-recapture models based on grizzly bear rub data from northwest Montana, USA.

Model	Female				Male	
	1	2	3	4	1	3
**λ**	**1.050**	**1.050**	**1.042**	**1.043**	**1.033**	**1.031**
SE	0.020	0.020	0.018	0.018	0.013	0.014
LCI	1.010	1.010	1.006	1.006	1.007	1.007
UCI	1.089	1.090	1.079	1.079	1.060	1.060

Detections used in these estimates excluded one outlier detection of a female in 2010. These estimates use the delta method to incorporate standard error, SE. Lower and upper confidence intervals, LCI and UCI, are 95%.

## Discussion

Using genetic detection data from natural bear rub sites in an SCR modeling framework, we estimated ecosystem-scale as well as local trends in a recovering grizzly bear population. Grizzly bear numbers in northwestern Montana were extremely low in the early 1900s^43^. Park protection and high-quality habitat resulted in recovery occurring earlier in Glacier NP than in the rest of the ecosystem^8^. The annual density surfaces produced by our model graphically depict the growth of the population outside of Glacier NP and how bear distribution changed 2004–2012. As the population expanded from the core in-and-adjacent-to Glacier NP, the population filled in much of the southern two thirds of the NCDE where few or no bears were found at the start of our study (Fig. 2). While the population as a whole grew at an annual rate of 1.043 in 2004–2012, growth rates were highest south of US 2 where initial density was low (Fig. 3). Population density remained fairly stable in Glacier NP and on the Blackfeet Reservation east of the Park 2004–2012 where 2004 densities were highest.

Because the use of SCR to model population trend surfaces from genetic detection data is new, we confirmed that independent estimates and ancillary data were consistent with patterns we found in population density and growth rates. Our annual rate of change for females 2004–2009 was similar to a previously published estimate for the NCDE (λ = 1.031 (95% CI: 0.928–1.102)) made using female vital rates derived from telemetry data and a known-fate estimator^20^. Our growth rate was also similar to other recovering brown bear populations (see Supplementary Information online for details). As the population grew, *N*_*e*_ in the NCDE also increased between 2004 and 2012 and generally tracked population trend^39^. The number of detections as well as the number of immigrants (based on a parentage assessment) consistently increased between 2004 and 2012 in the areas with the highest growth rates and lowest initial density, i.e. central, southern, and eastern regions^44^. These same areas, which exhibited lower density and diversity in 2004 (Fig. 2) also increased in genetic diversity^8,44^. This is consistent with previous evidence that bears south of US 2 were split into several small semi-isolated groups^8,44^. We also would expect to see bears moving into habitat that was not at carrying capacity as the population grew.

Our SCR models were highly sensitive to a spatial outlier. Even though our dataset was larger than most where SCR has been applied, we found that inclusion of a single extended movement changed the top models, evaluated through AIC, versus when the outlier was removed (Table S3 online, Table 3). We fit models without this point because such a large movement within one year is extremely uncommon for a female grizzly bear and resulted in unrealistically large home range and σ estimates. Removal of outliers from spatial data where the goal is fitting a declining function (e. g. half normal) is common. For example, in distance sampling^42^ it is common to remove outliers to allow fitting a simpler model^45^. This often leads to a fairly heavy right truncation (e. g. 5%), and in some cases left truncation, that is normative in distance modeling^46–48^. The treatment of space in distance modeling conceptually matches its treatment in SCR modeling, and some level of right truncation may be appropriate. In our case, while the decision to remove the outlier affected the best models and some detection and density estimates, our primary conclusions–that the population was growing and that density was increasing fastest in the southern portion of the ecosystem–were robust. However, we have no reason to believe that this is a generalizable conclusion. Our results were consistent with Stenhouse *et al*.^35^ in finding SCR estimates to be sensitive to an uncommon extreme movement. While SCR should be robust to common large movements^36^, we suggest comparing results with and without extreme movements to assess the impacts on all parameters.

We have seen no discussion of this sensitivity in the SCR literature and, in distance sampling, the arguments presented to support right truncation are primarily based on practicality rather than theoretical optimization^45^. Lacking any guidance in the literature we have presented results from analyses with and without the outlier, but have concentrated on the truncated dataset because it is likely more representative of the population. We believe that this issue requires additional study and that the field probably should develop rules for right truncation similar to those adopted in the distance sampling literature. Incorporation of telemetry might be used to reduce the effect of outliers, but this requires further study.

We hypothesize that non-spatial sources of heterogeneity of detection at rubs could bias density estimates. Females may limit rubbing in non-breeding years to avoid males and thereby protect dependent young. However, the proportion of females in our sample was very similar in all years except 2009 when sampling effort and baseline female capture probability were lowest. Our models accounted for heterogeneity in individual detection in space, because we believe that to be the largest source of heterogeneity. However, we could not model other potential sources of individual heterogeneity. For instance, we could not assign a history of human-bear conflict or breeding status to individuals, both of which could cause avoidance of the anthropogenic routes we sampled along and result in depressed detection rates. Nonetheless, as long as the proportion of the detectable population sampled is constant among years this approach provides a valid estimate of the trend for the population. We expect that the biases should be similar across years, which means that our trend estimates are reliable even if there is bias in density estimates. See Supplementary Information online for discussion of sampling efficiency at rub sites.

One of the most appealing aspect of using rub trees and SCR to detect trends is that both the data and the analyses are spatially explicit. Other analyses (e. g. known-fate^49,50^) do not consider the location of captures and recaptures, and thus require evenly and consistently distributed data collection^51^. Rub trees provide a huge spatial sample of individual bears at minimal cost. This allows patterns such as the infilling and range expansion we observed in this study to be directly observed. Combined with SCR analyses, the resulting density maps allow the detailed evaluation of where and when the population is increasing or declining which, in turn, permits direct checks of the data. Boulanger *et al*.^52^ similarly used SCR and density surface modelling to test whether bear population density was related to habitat value, mortality risk, or a combination of factors across the range of occupied grizzly bear habitat in Alberta, Canada. A population that is expanding based on population-wide changes in density may expand its range and increase its density in areas previously minimally occupied. We were able to demonstrate these relationships, providing increased confidence that the density shifts were appropriately related to actual population dynamics. Further, areas of population growth or decline can be linked to spatial covariates such as habitat quality or human population densities to evaluate the proximal causes of these population changes.

Fine scale information on changes in density has great utility for adaptive management, prioritizing management to preserve animal migrations^31^, and evaluating interspecific competition^53^ and resource selection^29^. Here we have used multiple density surfaces to assess infilling of a grizzly population in areas with previously low density. This was possible because our sample sites were distributed at a fine scale (spacings < 3 σ in all years) across our area of inference (Fig. 1b). We are confident of change at this coarse resolution, but suggest caution in over-interpreting change at the high resolution (16 km^2^) scale, because no estimates of variance are currently possible at that scale and little research has evaluated the appropriate resolution for estimating changes in density for animals with large versus small home ranges.

Changes in population status can occur fairly quickly in a recovering population even for species with low reproductive rates such as grizzly bears^44^. Informed management of forest carnivore populations requires insight into temporal and spatial variation in density and growth rates, particularly when large local declines can rapidly occur^54^. Sampling populations, such as ours, that occupy vast, remote areas in heterogeneous landscapes and monitoring during range expansion is especially challenging. Our genetic detection sampling approach coupled with SCR modeling allowed us to estimate differential growth rates of an expanding grizzly bear population and provided insight into how those patterns developed. For populations experiencing range expansion and thus violating the assumption of geographic closure, the SCR methods we employed are ideal because unlike non-spatial models, they are able to estimate the area to which the population estimates apply. While our dataset was so large that it restricted the set of methods available to us due to computational requirements, recent developments in open SCR models will additionally provide information on recruitment and survival^55,56^. Knowledge of spatial variation in trend can be used to design monitoring and management strategies tailored to area-specific needs. Spatial patterns revealed by our approach may identify source and sink habitats or ecological traps that merit enhanced management focus. Finally, the value of obtaining ancillary information on metrics such as gene flow, genetic diversity, population structure, the number of breeders, and fitness from monitoring population trend makes a compelling case for including a genetic sampling approach in monitoring programs.

## Supplementary information

Supplemetary information

## Data Availability

The genetic profiles have been deposited in ScienceBase and are publicly available^57^. Because grizzly bears are a US Government Endangered Species Act-listed species and spatial detection data could be used to identify grizzly bear concentration areas, access to precise locations may be limited. Field data and computer code are available from US Geological Survey, Northern Rocky Mountain Science Center, West Glacier, Montana 59936 USA.
