# Supplementary material for: Using bear rub data and spatial capture-recapture models to estimate trend in a brown bear population
Source: Sci Rep. 2019 Nov 14;9:16804. doi: 10.1038/s41598-019-52783-5 (PMC6856102; doi:10.1038/s41598-019-52783-5)

Using bear rub data and spatial capture-recapture models to estimate trend in a brown bear population

Katherine C. Kendall, Tabitha A. Graves, J. Andrew Royle, Amy C. Macleod, Kevin S. McKelvey, John Boulanger, and John S. Waller

SUPPLEMENTARY INFORMATION

Introduction

Although bear populations are challenging to monitor, bears possess an attribute that facilitates sampling them. Bears routinely rub on trees and other objects vigorously, often leaving hair behind. The most likely explanation for this ubiquitous activity is that it is a form of marking behaviour and functions as chemical signaling to other bears[1]. This tendency to rub results in the deposition of hair from individual bears at multiple sites. Because bears often use trails to travel, rub objects along trail networks can be located visually and therefore represent entirely passive detection; the sampling process has no effect on bear behaviour. While bears with a previous history of capture and handling have lower detection rates at baited hair corrals than bears that have not been trapped[2], no such effect has been found at bear rubs[3].

Grizzly bear and black bear (*U. americanus*) marking behaviour is well documented, if imperfectly understood[4,5,6,7]. Bears of all sex and age classes rub throughout the active season with variable frequency[8]. Evidence of rubbing is found throughout occupied habitat off-trail as well as along maintained and wildlife trails and by roads. Bears do not appear to discriminate much in selecting objects to mark. Bear hair has been found on a wide variety of natural (e.g. trees of all sizes and taxa, stumps, rocks) and man-made objects (e.g. sign and fence posts, utility poles, bridges, cabins)[8,9,10]. Female and subadult grizzlies rarely rub during the breeding season (May—June), however, their detection probability increases after low rates in spring and approaches male rates by late summer[3,8,11,12].

Many studies have demonstrated that genetic detection through hair collection is a highly effective way to sample bear populations; hair collection from a grid of baited hair corrals has been used extensively to study bear population abundance throughout the world (e.g.[13,14,15,16,]). Kendall *et al*.[3,8] added hair collection at natural bear rub sites as a secondary sampling approach to a primary method using an array of baited hair corrals. Because there was only partial overlap in individuals detected at hair corrals and rubs, the addition of this second sampling approach increased sample coverage and population abundance estimate precision while abundance estimates themselves were not significantly changed[17]. Results of subsequent studies that used concurrent sampling at hair corrals and bear rubs also found that the addition of rub site sampling was a cost-effective way to increase sample size and estimate precision[11,15,18]. While detection rates per sample site are higher at baited hair corrals than at unbaited rub sites[3,8,17,19,20] it is possible to sample a similar or larger portion of the population at rub sites than at baited corrals[11,18]. Sampling at rubs can also be more cost effective than at

baited hair corrals. In most studies using both methods, more field crew time was put into sampling at baited corrals than at rubs even when rubs greatly outnumbered corrals. For example, the ratio of field crews assigned to hair corral vs. rub sampling was over 4:1, while the ratio of individuals detected at corrals and rubs ranged from 1:1 to 2.5:1 for the studies conducted by Kendall *et al*.[3,8,18]. However, the overall efficiency of rub tree sampling depends on monitoring higher numbers of rub trees to offset the lower per site sampling efficiency of rub trees[19,20]. Therefore, rub tree sampling is most viable in areas with greater access, such as maintained trails.

Methods

Study area

Approximately 85% of the Northern Continental Divide Ecosystem (NCDE) is in public ownership, where grizzly bear conservation is a management objective. It includes Glacier National Park (NP), parts of 4 National Forests (Flathead, Kootenai, Helena-Lewis and Clark, and Lolo), five Wilderness areas (Bob Marshall, Great Bear, Mission Mountain, Rattlesnake, and Scapegoat), three Wilderness study areas (Mount Hefty-Tuchuck, Ten Lakes, and Thompson-Seton), Bureau of Land Management, U.S. Fish and Wildlife Service, and State land, and approximately half of the Blackfeet and one-third of the Flathead Indian Reservations. Private lands comprising the remaining 15% of the NCDE occur chiefly along the eastern, southern and western peripheries, but the Clearwater and Swan River Drainages, which are interior to the NCDE, contain both extensive diffuse housing and several small towns. Waterton Lakes National Park, Alberta and Akamina-Kishinena Provincial Park and provincial Crown lands in British Columbia, Canada border the NCDE to the north.

The climate and vegetation of the NCDE are shaped by the uplifted fault-blocks which formed the Rocky Mountains and by Pleistocene era ice ages[21]. Higher elevations receive more precipitation than lower slopes and valleys with annual precipitation ranging from over 170 cm in the higher elevations on the west side of Glacier NP to less than 38 cm on the eastern border. The Pacific maritime-influenced climate west of the Continental Divide is moister and more temperate than the colder and drier continental climate on the eastern side. The rugged terrain creates an array of habitats from alpine tundra and subalpine forests to moist mountain valleys, foothill groves, and semiarid grasslands in the rain shadow of large mountain masses[22]. Vegetation is characterized by coniferous forest, shrub fields and alpine tundra in the mountains, mixed deciduous–coniferous woodlands and herbaceous meadows in the valleys, and aspen (*Populus tremuloides*) stands, prairie grasslands, and agricultural fields along the eastern boundary[3].

Field methods

To identify bear rubs, we closely examined objects that included one or more of the following characteristics; smoothed and/or discoloured bark and branch stobs, bare ground at the base of trees/poles, entrenched bear trails leading to rubs from maintained trails, and bear bite or claw marks. We attempted to get broad and even geographic distribution of areas searched for rubs and did not prioritize areas thought to have higher densities of bears. In lower elevation areas

where there were fewer trees for bears to rub on, utility poles and fence lines provided sampling opportunities. To prevent damage to pack stock and their cargo, we used double-stranded smooth wire on trees that appeared to have been bumped by horse packs. We separated the twisted ends of smooth wire slightly to make them more effective in snagging hair. We visited rubs at least twice annually. All hair deposited on the wire was collected during each visit. The hair found on rubs during the initial visit each year was not analyzed and did not contribute to detection histories because it could have been deposited the previous year. A flame was passed under each barb and wire after sample collection, and hair between wires was removed to prevent contamination when the next bear rubbed. All hairs from a 4-point barb or wire end were considered one sample.

Rubs were initially identified and set up in 2003 for sampling in 2004. Because we removed tags and wire on monitored rubs at the end of the 2004 field season, the rub survey network was re-established in 2009. The time required for initial set up meant that rubs in some remote areas were not set up until late summer 2009 or spring 2010, leading to lower rub sampling effort and extent in 2009 than in all other years.

Genetic analysis

In 2004, we attempted genetic analysis on all hair samples collected. During 2009—2012, we subsampled hair for analysis to limit genotyping costs. When we collected more than one hair sample during a visit to a rub, we genotyped the two samples with the most and/or largest follicles to maximize the amount of DNA in the sample. If sample quality was equal, we selected non-adjacent samples to increase the probability of detecting more than one individual per site. If there were distinct differences in hair colour between samples, we selected the best 2 samples from each, if available.

Spatial capture-recapture modeling

We included several trap covariates to describe detection. We calculated security level based on the location of a trap, where National Park Service =10, Forest Service =7, State and private forest lands =3, and all other =1. We used the standard deviation of curvature within a 250-m buffer of the trap covariate based on Ironside *et al.*[23], which found it to be the most robust measure of terrain ruggedness. We also evaluated the effects of percent canopy cover based on the cover layer from Landfire, using the most recent data for that year. We updated the 2004 layer to reflect reductions in canopy cover due to some logging and extensive fires between the 2000/2001 imagery and the 2004 dataset.

Results

Rub sampling effort

Sampling primarily occurred in forested habitats, however, bears' predilection to rub on fence and utility poles ensured sampling opportunities in prairie and other treeless habitats and agricultural areas. In 2004, search effort to identify rubs was lower along the eastern edge of our study area because there were few trails or little bear sign to guide our efforts. Sampling in this area was primarily in riparian corridors. Anecdotal information also indicated that few

bears ranged east of the Rocky Mountain foothills at that time. After 2004, we expanded the number of monitored rub objects along the eastern foothills and plains as bears began to reoccupy former range. Starting in 2009, additional rub objects on the eastern edge were, therefore, added to the monitoring network. Rubs continued to be identified and added as sampling sites during early 2010 and then they generally remained in our monitoring network for the duration through 2012. A small number (<1%) of trees blew down or could not be relocated during our study; however, other monitored rubs were typically < 1 km away and in some cases, nearby new rubs were added to the monitoring network to replace them. As a result, sampling distribution and effort was nearly identical 2010—2012.

Discussion

Our point estimate for the annual rate of change, 1.043, was fairly high for an interior grizzly/brown bear population. Stable growth rates ($\lambda \sim 1.0$) have been reported in the past for a number of interior populations in the Rocky Mountains of the United States and Canada (e.g. [24,25]). More recently, however, several populations have grown at rates comparable to those we found in the NCDE, likely a reflection of demographic recovery in response to conservation measures[26,27,28]. A study examining demographic mechanisms underpinning genetic assimilation of remnant groups of grizzly bears in the NCDE found that the increases in genetic diversity they identified resulted primarily from immigration[29]. It is likely that a combination of growth from within the remnant population coupled with bears moving in from higher density areas and the mathematics of small numbers in a local area (a change from 1 to 2 bears is a 100% increase) was responsible for the highest rates of pixel-specific growth we found in the NCDE (Figure 3). Quantifying local change rates using alternate measures such as gain or loss in density per pixel may be useful in smoothing this effect.

Bear species throughout the world exhibit rubbing behaviour; e.g., grizzly[30], European brown bear[31,32], Japanese brown bear[33], American black bears[34], and Asiatic black bears[15]. A growing number of studies have taken advantage of this behaviour to study bear demography (e.g., [8,11,35]), dispersal[29], and behaviour[7,36]. Developing a better understanding of which bears rub, why they rub, and where and when they do it will make this sampling method more powerful. The data obtained from genetic detection can inform multiple aspects of population status and mechanisms. For example, studies of habitat effects on abundance[38], connectivity and dispersal[38,39], the role of social learning[36], genetic assimilation in remnant subpopulations[29], and the relationship between effective and demographic population size in continuously distributed populations[40], were based on the genetic samples collected for this and earlier genetic detection studies in the NCDE[3,8].

Efficient sampling methods increase sample coverage while decreasing monitoring costs. The number of individual bears we sampled through hair collection at rub sites was an order of magnitude larger and more evenly spatially distributed, e.g. in the expansion area, than would typically be possible through live capture methods. Thus, we expect that genetic monitoring will be more sensitive to spatial and temporal changes in population demography than approaches that involve fewer individuals. Our genetic detection network yielded large sample

sizes and precise estimates of population growth rate with 5 years of rub detection data (2004, 2009—2012). Other approaches to obtaining similar spatial data, such as a grid of bear hair corrals, cost millions of dollars to implement across a region of this size[8]; repeated sampling to obtain trend estimates using these methods across areas as broad as the NCDE has to date proved unsustainable. It may be possible to monitor populations such as ours with adequate precision with less sampling effort and at lower cost than in this study. The use of multiple detection methods[17], clustered and stratified sampling designs[19,35], adding telemetry data, or integrated population models that combine SCR data with lower resolution information[41,42] could further reduce the sampling and, thus, expense of SCR-based studies in the future.

References

1. Clapham, M., Nevin, O. T., Ramsey, A. D. & Rosell, F. The function of strategic tree selectivity in chemical signalling of brown bears. *Animal Behaviour* **85**, 1351—1357 (2013).

2. Boulanger, J. *et al*. Use of occupancy models to estimate the influence of past live captures on detection probabilities of grizzly bears using DNA hair snagging methods. *Journal of Wildlife Management* **72**, 589–595 (2008).

3. Kendall, K. C. *et al*. Grizzly bear density in Glacier National Park, Montana. *Journal of Wildlife Management* **72**, 1693–1705 (2008).

4. Burst, T. L. & Pelton, M. R. Black bear mark tree in the Smokey Mountains. *International Conference on Bear Research and Management* **5**, 45—53 (1983).

5. Green, G. I. & Mattson, D. J. Tree rubbing by Yellowstone grizzly bears (*Ursus arctos*). *Wildlife Biology* **9**, 1–9 (2003).

6. Clapham, M., Nevin, O. T., Ramsey, A. D. & Rosell, F. Hypothetico-deductive approach to assessing the social function of chemical signalling in a non-territorial solitary carnivore. *PLoS ONE* **7**, e35404; 10.1371/journal.pone.0035404 (2012).

7. Clapham, M., Nevin, O. T., Ramsey, A. D. & Rosell, F. Scent-marking investment and motor patterns are affected by the age and sex of wild brown bears. *Animal Behaviour* **94**, 107—116; 10.1016/j.anbehav.2014.05.017 (2014).

8. Kendall, K. C. *et al*. Demography and genetic structure of a recovering grizzly bear population. *Journal of Wildlife Management* **73**, 3–17 (2009).

9. Kendall, K. C. *et al*. Density, distribution, and genetic structure of grizzly bears in the Cabinet-Yaak ecosystem. *Journal of Wildlife Management* **80**, 314—331 (2016).

10. Karamanlidis, A. A., Youlatos, D., Sgardelis, S. & Scouras, Z. Using power poles to document presence of bears in Greece. *Ursus* **18**, 54—61 (2007).

11. Sawaya, M. A., Stetz, J. B., Clevenger, A. P., Gibeau, M. L. & Kalinowski. S. T. Estimating grizzly and black bear population abundance and trend in Banff National Park using noninvasive genetic sampling. *PLoS ONE* **7**, 1–12; 10.1371/journal.pone.0034777 (2012).

12. Whittington, J., Hebblewhite, M. & Chandler, R. B. Generalized spatial mark–resight models with an application to grizzly bears. *Journal of Applied Ecology* **55**, 157—168 (2018).

13. Woods, J. G. *et al*. Genetic tagging of free-ranging black and brown bears. *Wildlife Society Bulletin* **27**, 616—627 (1999).

14. Taberlet, P. *et al*. Noninvasive genetic tracking of the endangered Pyrenean brown bear population. *Molecular Ecology* **6**, 869—876 (1997).

15. Latham, E., Stetz, J. B., Seryodkin, I., Miquelle, D. & Gibeau, M. L. Non-invasive genetic sampling of brown bears and Asiatic black bears in the Russian Far East. *Ursus* **23**, 145—158 (2012).

16. Murphy, S. M. *et al*. Characterizing recolonization by a reintroduced bear population using genetic spatial capture-recapture. *Journal of Wildlife Management* **80**, 1390—1407 https://doi.org/10.1002/jwmg.21144 (2016).

17. Boulanger, J. *et al*. Use of multiple data sources to improve DNA-based mark-recapture population estimates of grizzly bears. *Ecological Applications* **18**, 577–589 (2008b).

18. Kendall, K. C. *et al*. Density, distribution, and genetic structure of grizzly bears in the Cabinet-Yaak ecosystem. *Journal of Wildlife Management* **80**, 314—331 (2016).

19. Stenhouse, G. B. *et al*. Estimates of grizzly bear population size and density for the 2014 Alberta Yellowhead Population Unit (BMA 3) and south Jasper National Park Inventory Project. Report prepared for Weyerhaeuser Ltd., West Fraser Mills Ltd, Alberta Environment and Parks, and Jasper National Park. https://friresearch.ca/resource/estimates-grizzly-bear-population-size-and-density-final-report (2015).

20. Lamb, C. T., Walsh, D. A. & Mowat. G. Factors influencing detection of grizzly bears at genetic sampling sites. *Ursus* **27**, 31—44 (2016).

21. Alt, D. D. & Hyndman, D. W. Roadside geology of the northern Rockies. Mountain Press Publishing Company, Missoula, MT (1972).

22. Arno, S. F. Forest Regions of Montana. USDA Forest Service Research Paper INT-218, Intermountain Forest and Range Experiment Station, Forest Service, U. S. Department of Agriculture, Ogden, UT 84401 (1979).

23. Ironside, K.E., Mattson, D.J., *et al*. Geomorphometry in Landscape Ecology: Issues of scale, physiography, and application.   Environment and Ecology Research 6(5): 397-412 (2018). Doi: 10.13189/eer.2018.060501

24. Wielgus, R. B., Bunnell, F. L., Wakkinen, W. L., & Zager, P. E. Population dynamics of Selkirk Mountain Grizzly Bears. *J. Wildlife Management* **58**, 266—272 (1994).

25. Mace, R. D. & Waller, J. S. Demography and population trend of grizzly bears in the Swan Mountains, Montana. *Conservation Biology* **12**, 1005—1016 (1998).

26. Kindberg, J. *et al*. Estimating population size and trends of the Swedish brown bear Ursus arctos population. *Wildlife Biology* **17**, 114—123 (2011).

27. Garshelis, D. L., Gibeau, M. L. & Herrero, S. Grizzly bear demographics in and around Banff National Park and Kananaskis Country, Alberta. *Journal of Wildlife Management* **69**, 277—297 (2005).

28. Morehouse, A. T. & Boyce, M. S. Grizzly bears without borders: Spatially explicit capture-recapture in southwestern Alberta. *Journal of Wildlife Management* **80**, 1152—1166; 10.1002/jwmg.21104 (2016).

29. Mikle, N., Graves, T. A., Kovach, R., Kendall, K. C. & Macleod, A. C. Demographic mechanisms underpinning genetic assimilation of remnant groups of a large carnivore. *Proc. Royal Society B* **282**; 10.1098/rspb.2016.1467 PMID: 27655768 (2016).

30. Kendall, K. C., Metzgar, L. H., Patterson, D. A. & Steele, B. M. Power of sign surveys to monitor population trends. *Ecological Applications* **2**, 422—430 (1992).

31. Karamanlidis, A. A. *et al*. Noninvasive genetic studies of brown bears using power poles. *European Journal of Wildlife Research* **56**, 693—702 (2010).

32. De Barba, M. *et al*. 2010. Comparing opportunistic and systematic sampling methods for non-invasive genetic monitoring of a small translocated brown bear population. *Journal of Applied Ecology* **47**, 172—181 (2010).

33. Sato, Y. *et al*. Selection of rub trees by brown bears (*Ursus arctos*) in Hokkaido, Japan. *Acta Theriologica* **59**, 129—137; 10.1007/s13364-013-0143-z (2014)

34. Burst, T. L. & Pelton, M. R. Black bear mark tree in the Smokey Mountains. *International Conference on Bear Research and Management* **5**, 45—53 (1983).

35. Humm, J. M., McCowen, J. W., Scheick, B. K., & Clark, J. D. Spatially explicit population estimates for black bears based on cluster sampling. *Journal of Wildlife Management* **81**, 1187—1201. https://doi: 10.1002/jwmg.21294 (2017).

36. Morehouse, A.T., Graves, T. A., Mikle, N. & Boyce, M. S. Nature vs. nurture: Evidence for social learning of conflict behavior in grizzly bears. *PLoS ONE* **11**, e0165425; 10.1371/journal.pone.0165425 (2016).

37. Graves T. A. *et al*. Balancing precision and risk: Should multiple detection methods be analyzed separately in N-mixture models? *PLoS ONE* **7**; 10.1371/journal.pone.0049410 (2012).

38. Graves, T. A., Chandler, R. B., Royle, J. A., Beier, P. & Kendall, K. C. Estimating landscape resistance to dispersal. *Landscape Ecology* **29**, 1201—1211 (2014).

39. Dilkina, B. *et al*. Trade-offs and efficiencies in optimal budget-constrained multispecies corridor networks. *Conservation Biology* **31**, 192—202; 10.111/cobi.12814 (2017).

40. Pierson, J. C., Graves, T. A., Banks, S. C., Kendall, K. C. & Lindenmayer, D. B. Relationship between effective and demographic population size in continuously distributed populations. *Evolutionary Applications* **11**, 1162—1175; 10.1111/eva.12636 (2018).

41. Schaub. M. & Abadi, F. Integrated population models: a novel analysis framework for deeper insights into population dynamics. *Journal of Ornithology* **152** (Supplement 1): S227—S237 (2011).

42. Chandler, R. B. & Clark, J. D. Spatially explicit integrated population models. *Methods in Ecology and Evolution* **5**, 1351—1360 (2014).

43. U.S. Geological Survey. USGS National Elevation Dataset (NED) 1 arc-second Downloadable Data Collection from The National Map 3D Elevation Program (3DEP) - National Geospatial Data Asset (NGDA) National Elevation Data Set (NED): U.S. Geological Survey, https://www.sciencebase.gov/catalog/item/5825a0c3e4b01fad86db66dc (2016).

Table S1. Description of full set of candidate models developed to estimate grizzly bear population density using spatial capture recapture data from northwestern Montana, USA. Spatial detection histories were developed through genetic identification of individual bears from hair collected at natural bear rub sites. Model 17 (= Model 8 + a term for translocated bears) and Model 18 (= Model 16 + translocations) did not converge because few individuals were identified as having been translocated in our dataset.

Model[a]	D	p_0	σ
1	~ linear	~ year + dur + jul + jul2 + beh + cover + cover2 + curv + sec	~ year
2	~ linear	~ year + dur + jul + beh + cover + cover2 + curv + sec	~ year
3	~ year	~ year + dur + jul + jul2 + beh + cover + cover2 + curv + sec	~ year
4	~ year	~ year + dur + jul + beh + cover + cover2 + curv + sec	~ year
5	~ year	~ dur + jul + beh + cover + cover2 + curv + sec	~ 1
6	~ year	~ dur + jul + beh + cover + cover2 + curv + sec	~ year
7	~ year	~ dur + jul + jul2 + beh + cover + cover2 + curv + sec	~ 1
8	~ year	~ dur + jul + jul2 + beh + cover + cover2 + curv + sec	~ year
9	~ year	~ year + dur + jul + beh + cover + cover2 + curv + sec	~ 1
10	~ year	~ year + dur + jul + jul2 + beh + cover + cover2 + curv + sec	~ 1
11	~ linear	~ dur + jul + beh + cover + cover2 + curv + sec	~ 1
12	~ linear	~ dur + jul + beh + cover + cover2 + curv + sec	~ year
13	~ linear	~ dur + jul + jul2 + beh + cover + cover2 + curv + sec	~ 1
14	~ linear	~ dur + jul + jul2 + beh + cover + cover2 + curv + sec	~ year
15	~ linear	~ year + dur + jul + beh + cover + cover2 + curv + sec	~ 1
16	~ linear	~ year + dur + jul + jul2 + beh + cover + cover2 + curv + sec	~ 1

[a] Model notation:

D	density (bears/1,000 km^2)
p_0	baseline detection probability
σ	sigma; spatial scale parameter related to the amount of space used by each individual
~	"a function of"
year	parameter is year-specific
linear	density is a linear function of time
dur	duration of sampling interval; number of days since previous sampling visit
jul	Julian day; linear effect of season on detection probability
jul2	Julian day squared; quadratic effect of season on detection probability
beh	behavioural response; a visit of some bear at some bear rub changes its subsequent probability of detection at that rub
cover	percent of mean canopy cover within a 250 m radius of the trap cover2 percent of mean canopy cover squared
curv	standard deviation of terrain curvature within a 250 m radius of the trap sec security level as determined by land ownership policies at the trap: 10=NPS, 7=FS, 3= state/other, public, 1=private)

Table S2. Female and male parameter estimates (untransformed) for the most supported models (Table 3) developed to estimate trend 2004—2012 in a grizzly bear population using spatial capture-recapture data from northwestern Montana, USA.

	Female			Male		
Parameter[a]	Estimate	SE	P(>|z|)	Estimate	SE	P(>|z|)
D_0.(Intercept)	-1.891	0.05	0	-2.25	0.04	0
D.beta.Trend	0.048	0.019	0.011	0.033	0.013	0.011
p_0.(Intercept)	-5.3	0.376	0	-5.079	0.106	0
p_0.2009	-1.092	0.269	0	0.647	0.119	0
p_0.2010	0.508	0.215	0.018	0.104	0.115	0.365
p_0.2011	0.217	0.217	0.317	0.281	0.118	0.017
p_0.2012	-0.014	0.211	0.946	-0.039	0.114	0.735
p.beh	1.456	0.184	0	1.589	0.12	0
p.dur	0.476	0.1	0	0.51	0.06	0
p.jul	0.542	0.217	0.012	0.124	0.039	0.002
p.jul2	-0.059	0.033	0.075	-0.05	0.007	0
p.cover	-0.207	0.068	0.002	-0.126	0.042	0.002
p.cover2	-0.103	0.058	0.077	-0.022	0.034	0.505
p.curv	0.438	0.124	0	-0.436	0.081	0
p.sec	0.236	0.067	0	0.458	0.047	0
σ.(Intercept)	1.481	0.053	0	2.23	0.027	0
σ.2009	0.284	0.096	0.003	-0.236	0.04	0
σ.2010	-0.367	0.069	0	-0.014	0.04	0.724
σ.2011	-0.304	0.072	0	-0.199	0.041	0
σ.2012	-0.13	0.07	0.062	-0.02	0.041	0.632

[a] Parameter notation:

D	density (bears/1,000 km^2)
p_0	baseline detection probability in 2004
p_0.2009-12	year-specific detection probability
p.beh	behavioural response; a visit of some bear at some bear rub changes its subsequent probability of detection at that rub
p.dur	duration of sampling interval; days since previous sampling visit
p.jul	Julian day; linear effect of season on detection probability
p.jul2	Julian day squared; quadratic effect of season on detection probability
p.cover	proportion of canopy cover
p.cover2	proportion of cover squared
p.curv	standard deviation of terrain curvature
p.sec	mean security level as determined by land ownership
σ	sigma; parameter related to the amount of space used by each individual
σ.2009-12	year-specific sigma

Table S3. Model selection results for females including all detections, i.e. **including one additional detection 190 km from the other detections of that bear not included in the models shown in Table 3**. Spatial covariates (spatial covs= cover, cover2, curv) were calculated within a 250-m radius of each rub sampling site. Only models with cumulative weights up to 1 are presented.

Females - with outlier point						
Model	D	p_0	σ	ΔAIC	Weight	CumWt
3	~year	~year+dur+jul+jul2+beh+spatial covs	~year	0.00	0.51	0.51
4	~year	~year+dur+jul+beh+spatial covs	~year	0.15	0.47	0.98
1	~linear	~year+dur+jul+jul2+beh+spatial covs	~year	7.87	0.01	0.99
2	~linear	~year+dur+jul+beh+spatial covs	~year	7.92	0.01	1.00

D	density (bears/1,000 km^2)
*p*0	baseline detection probability
σ	sigma; spatial scale parameter related to the amount of space used by each individual
ΔAIC	cumulative change in Akaike Information Criterion
Weight	measure of support for each model
CumWt	cumulative measure of support for the models
~	"a function of"
linear	density is a linear function of time
year	parameter is year-specific
dur	duration of sampling interval; number of days since previous sampling visit
jul	Julian day; linear effect of season on detection probability
jul2	Julian day squared; quadratic effect of season on detection probability
beh	behavioural response; a visit of some bear at some bear rub changes its subsequent probability of detection at that rub
spatial covs	spatial covariates:
cover	percent of mean canopy cover
cover2	percent of mean canopy cover squared
curv	standard deviation of terrain curvature
sec	security level as determined by land ownership policies at the trap: 10=National Park Service, 7=Forest Service, 3= state/other public, 1=private)

Table S4. Female parameter estimates (untransformed) for the most supported models (Table S3) using all detections, i.e. **including one additional detection 190 km from the other detections of that bear not included in the models shown in Tables 3 and 4**. Estimates for model 4 were nearly identical to model 3 and estimates for model 2 were nearly identical to model 1, so are not shown.

Females - with outlier point					
Model 3			Model 1		
Parameter[a]	Estimate	SE	Parameter[a]	Estimate	SE
D_0.(Int)	-2.048	0.108	D_0.(Int)	-2.000	0.054
D.beta.2009	0.049	0.180	D.beta.linear	0.034	0.017
D.beta.2010	-0.171	0.143			
D.beta.2011	0.271	0.147			
D.beta.2012	0.326	0.141			
p_0.(Int)	-5.268	0.373	p_0.(Int)	-5.224	0.372
$p_0$2009	-1.010	0.281	p_0.2009	-1.064	0.267
p_0.2010	-1.641	0.21	p_0.2010	-1.764	0.209
p_0.2011	0.195	0.219	p_0.2011	0.207	0.216
p_0.2012	-0.020	0.211	p_0.2012	-0.009	0.209
p.beh	1.838	0.186	*p*.beh	1.813	0.186
p.dur	0.482	0.097	*p*.dur	0.485	0.097
p.jul	0.476	0.215	*p*.jul	0.469	0.214
p.jul2	-0.047	0.033	*p*.jul2	-0.046	0.033
p.cover	-0.209	0.064	*p.cover*	-0.208	0.064
p.cover2	-0.123	0.057	*p*.cover2	-0.121	0.057
p.curv	0.442	0.121	*p*.curv	0.438	0.121
p.sec	0.256	0.064	*p*.sec	0.249	0.064
σ.(Int)	1.492	0.054	σ.(Int)	1.497	0.054
σ.2009	0.286	0.098	σ.2009	0.278	0.097
σ.2010	0.890	0.072	σ.2010	0.856	0.070
σ.2011	-0.303	0.073	σ.2011	-0.299	0.073
σ.2012	-0.134	0.071	σ.2012	-0.127	0.071

[a] Parameter notation:

D_0.(Int)	density (bears/1,000 km^2) (Intercept)
p_0.(Int)	baseline detection probability in 2004 (Intercept)
p_0.2009-12	year-specific detection probability
p.beh	behavioral response; a visit of some bear at some bear rub changes its subsequent probability of detection at that rub
p.dur	duration of sampling interval; days since previous sampling visit
p.jul	Julian day; linear effect of season on detection probability
p.jul2	Julian day squared; quadratic effect of season on detection probability
p.cover	proportion of canopy cover

p.cover2	proportion of cover squared
p.curv	standard deviation of terrain curvature
p.sec	mean security level as determined by land ownership
σ.(Int)	baseline sigma (Intercept); related to the amount of space used by each individual
σ.2009-12	year-specific sigma

Table S5. Point estimates (transformed) of female density (bears/1,000 km^2), spatial scale of movement (σ), and baseline detection probability (p_0) parameters for models developed to estimate grizzly bear population trend using spatial capture-recapture data from the Northern Continental Divide Ecosystem in northwestern Montana, USA. a. **Estimates that include one outlier detection of a single female in 2010 that was 190 km distant from other detections of that individual.** b. Estimates that exclude the outlier detection point. The baseline detection probability reported here is for b=0, initial behavior covariate (prior to the first detection), and all other covariates at their mean level. When the outlier was included, the best model had year-specific density (model #3) versus a linear yearly trend in density (model #1) when the outlier point was excluded.

a. Females – with outlier point. Best model is #3

	Density		σ		p_0	
Model	3	1	3	1	3	1
2004	8.06	7.37	4.45	4.47	0.005	0.005
2009	8.47	8.75	5.92	5.90	0.002	0.002
2010	6.79	9.06	10.83	10.51	0.001	0.001
2011	10.57	9.38	3.29	3.31	0.006	0.007
2012	11.17	9.71	3.89	3.94	0.005	0.005

b. Females – excluding outlier point. Best model is #1

	Density		σ		p_0	
Model	3	1	3	1	3	1
2004	7.92	7.77	4.39	4.40	0.005	0.005
2009	8.39	9.90	5.89	5.84	0.002	0.002
2010	10.16	10.40	3.04	3.05	0.008	0.008
2011	10.39	10.91	3.24	3.24	0.006	0.006
2012	11.04	11.45	3.86	3.86	0.005	0.005

Figure S1. Locations of grizzly bear detections 2004, 2009—2012 (N=1,539) derived from sampling at natural bear rub sites in the Northern Continental Divide Ecosystem in northwestern Montana, USA. Maps were created from the USGS National Elevation Dataset[43] using ArcMap 10.2.

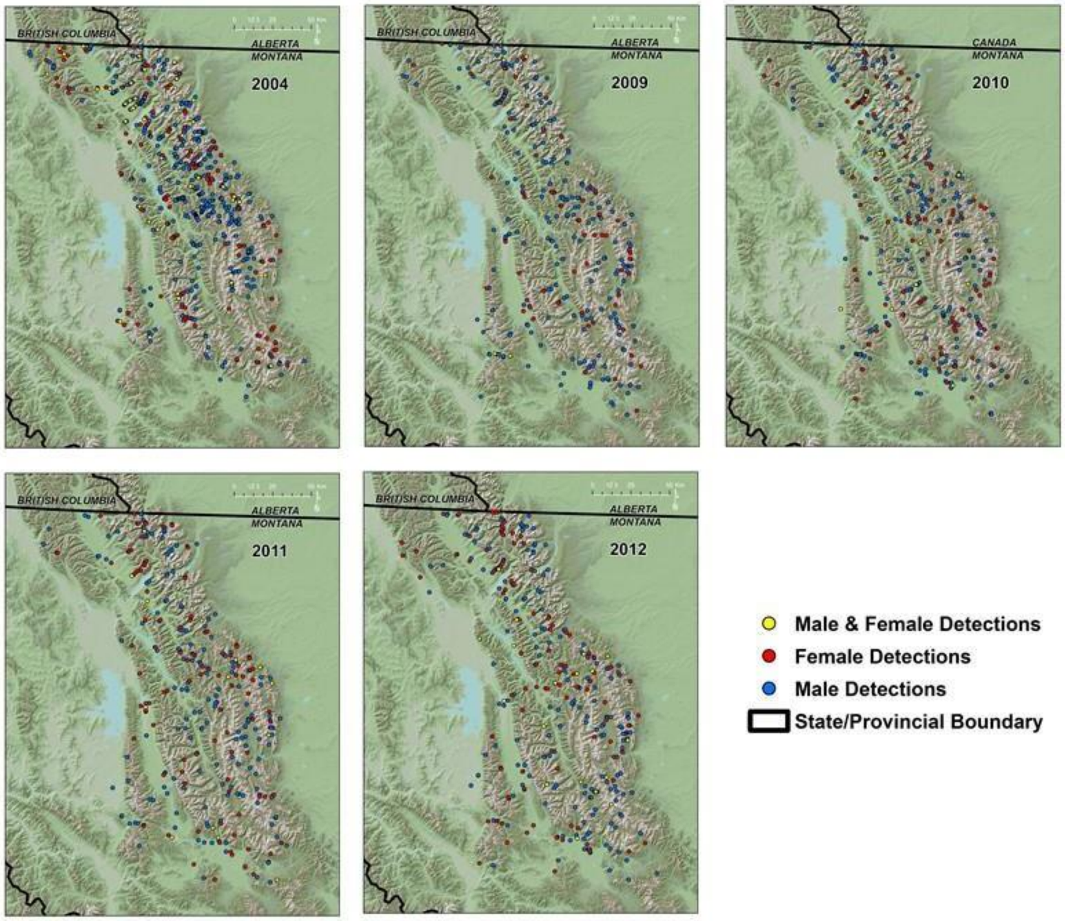

Supplement: Supplementary file 1 — Supplemetary information [file 41598_2019_52783_MOESM1_ESM.pdf]
